# Synthesis, Structure and Cytotoxic Activity of Mono- and Dialkoxy Derivatives of 5,8-Quinolinedione

**DOI:** 10.3390/molecules21020156

**Published:** 2016-01-27

**Authors:** Monika Kadela, Maria Jastrzębska, Ewa Bębenek, Elwira Chrobak, Małgorzata Latocha, Joachim Kusz, Maria Książek, Stanisław Boryczka

**Affiliations:** 1 Department of Organic Chemistry, School of Pharmacy with the Division of Laboratory Medicine in Sosnowiec, Medical University of Silesia in Katowice, 4 Jagiellońska Str., 41-200 Sosnowiec, Poland; ebebenek@sum.edu.pl (E.B.); echrobak@sum.edu.pl (E.C.); boryczka@sum.edu.pl (S.B.); 2 Department of Solid State Physics, Institute of Physics, University of Silesia, 4 Uniwersytecka Str., 40-007 Katowice, Poland; maria.jastrzebska@us.edu.pl (M.J.); maria.ksiazek@us.edu.pl (M.K.); 3 Silesian Center for Education and Interdisciplinary Research, University of Silesia, 75 Pułku Piechoty 1, 41-500 Chorzów, Poland; 4 Department of Cell Biology, School of Pharmacy with the Division of Laboratory Medicine in Sosnowiec, Medical University of Silesia in Katowice, 8 Jedności Str., 41-200 Sosnowiec, Poland; mlatocha@sum.edu.pl; 5 Department of Physics of Crystals, Institute of Physics, University of Silesia, 4 Uniwersytecka Str., 40-007 Katowice, Poland; joachim.kusz@us.edu.pl

**Keywords:** alkoxy-5,8-quinolinedione, crystal structure, cytotoxic activity

## Abstract

A series of 5,8-quinolinedione derivatives containing one or two alkoxy groups was synthesized and characterized by ^1^H- and ^13^C-NMR, IR and MS spectra. X-ray diffraction was used to investigate the crystal structures of 6-chloro-7-(2-cyjanoethoxy)-5,8-quinolinedione and 6,7-di(2,2,2-trifloroethoxy)-5,8-quinolinedione. All studied compounds were tested *in vitro* for their antiproliferative activity against three human cancer cell lines and human normal fibroblasts. Most of the compounds showed higher cytotoxicity than the starting compound, 6,7-dichloro-5,8-quinolinedione, and cisplatin, which was used as a reference agent.

## 1. Introduction

Cancer diseases are nowadays one of the most important health problems in the world. According to a World Health Organization report published in 2012, more than 14.1 million new cancer cases were diagnosed and about 8.2 million cancer-related deaths were recorded. The increase in cancer incidents is most likely associated with aging of population and unhealthy lifestyle including physical inactivity, smoking and poor diet.

**Figure 1:**
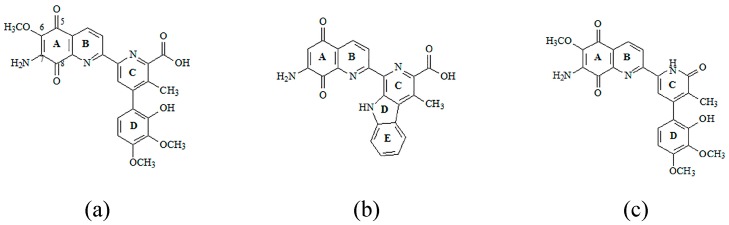
Structures of 5,8-quinolinedione antibiotics: (**a**) Streptonigrin; (**b**) Lavendamycin; and (**c**) Steptonigrone.

Antibiotics containing the 5,8-quinolinedione moiety, like Streptonigrin, Lavendamycin and Steptonigrone, are known for their high anticancer, antibacterial, and antiviral activities (Figure 1) [2,3,4].

In the 1970s, the anticancer activity of the Streptonigrin against leukemia cell lines was recognized to be one of great interest to researchers. However, because of its toxic side effects, a clinical trial was ended in 1977 [5,6] The structure–activity relationship of 5,8-quinolinedione antibiotics have been studied over the years. The research has shown that the most important part of the molecule is the 5,8-quinolinedione moiety, which is responsible for inhibiting DNA and RNA synthesis, and interfering with topoisomerase II action.

Moreover, 5,8-quinolinedione can be reduced by nicotinamide adenine dinucleotide phosphate(NAD(P)H) as a cofactor to form semiquinone or hydroquinone intermediates [7,8,9,10]. These compounds can react with oxygen yielding a regenerated 5,8-quinolinedione fragment and creating the hydroxyl radicals that are ultimately responsible for DNA strand cleavage. Furthermore, it was also found that the introduction of amine, hydroxyl or thiol substituents at position 6 or 7 of the 5,8-quinolinedione moiety results in an enhanced biological activity [3,4,11,12,13,14].

There are many reports on the synthesis and biological activity of the amine derivatives of 5,8-quinolinedione, whereas studies on the alkoxy analogues are rather scarce in the literature [9,15].

In the present work, we report the synthesis of mono- and disubstituted alkoxy derivatives of 5,8-quinolinedione and the structural characterization of some alkoxy compounds. All newly synthesized compounds were tested for cytotoxic activity against cancer cell lines such as human melanoma (C-32), glioblastoma (SNB-19), and breast cancer (MDA-MB-231) as well as normal fibroblasts (HFF-1) in order to obtain more information about the influence of one or two alkoxy groups on the anticancer activity of the compounds under study.

## 2. Results and Discussion

### 2.1. Chemistry

The starting compound, 6,7-dichloro-5,8-quinolinedione **1**, was obtained from 8-hydroxyquinoline. The chemical structure of **1** was determined using the proton nuclear magnetic resonance (^1^H-NMR), the carbon nuclear magnetic resonance (^13^C-NMR) and the infrared spectroscopy (IR). The band assignment of the spectra was done using literature data [16].

**Scheme 1:**
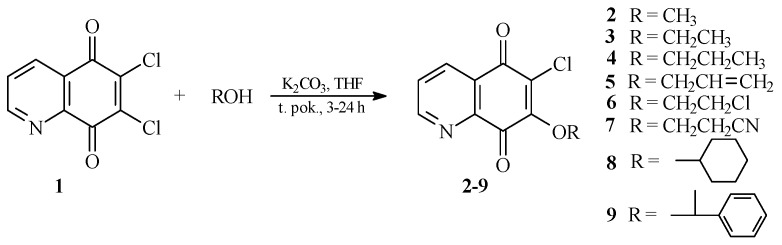
Synthesis of the 7-substituted derivatives of 5,8-quinolinedione, **2**–**9**.

As was previously described by Jastrzębska *et al.* [17], reaction of compound **1** with an amine group carried out in tetrahydrofuran (THF) and in the presence of potassium carbonate, gives two isomeric products. The major product of this is a compound containing an amino substituent at the 7 position [17]. In this study, the alkoxy derivatives of 5,8-quinolinedione were prepared using the same reaction conditions. The reactions of 6,7-dichloro-5,8-quinolinedione **1** with alcohols were performed in tetrahydrofuran in the presence of potassium carbonate giving products **2**–**9** (Scheme 1). After purification by column chromatography, derivatives **2**–**9** were obtained with yields of 53%–88%. Structures of all new compounds were confirmed based on ^1^H-, ^13^C-NMR, IR, electron ionization mass spectrometry (EI-MS) and high-resolution mass spectrometry (HR MS) methods.

In order to confirm the regioselectivity of the substitution reaction, ^13^C-NMR spectroscopy and X-ray diffraction analyses were performed. According to literature data [16], the ^13^C-NMR spectra of aliphatic amine derivatives show low intensity signals of C7, C6 and C8 atoms for the 6-substituted isomer, while those of C5, C6 and C7 atoms are of low intensity for the 7-substituted derivative. Therefore, the signal intensity of C5 and C8 atoms should be different depending on the position of the substituent. For the described products **2**–**9**, the signal of the C5 atom is of lower intensity than the signal of C8 atom. Therefore, it has been found that the compounds **2**–**9** contain the alkoxy group at the 7 position. Moreover, the X-ray diffraction analysis confirmed that **7** is a 7-substituted derivative.

In the literature [18], the nucleophilic substitution occurring at the C6 or C7 position was assumed to follow the S_N_2 mechanism. It was found that the solvent plays an important role in the reaction of 6,7-dichloro-5,8-quinolinedione **1** with amines and arylthiols. The 7-substituted derivative was the major product of the reaction when an aprotic solvent, like THF or dimethylformamide (DMF), was used. The reaction carried out in protic solvent such as water, led to 6-substituted derivatives with higher yields [17,18], whereas 6,7-dithioarylo compounds were synthesized when ethanol was used as a solvent [19].

**Scheme 2:**
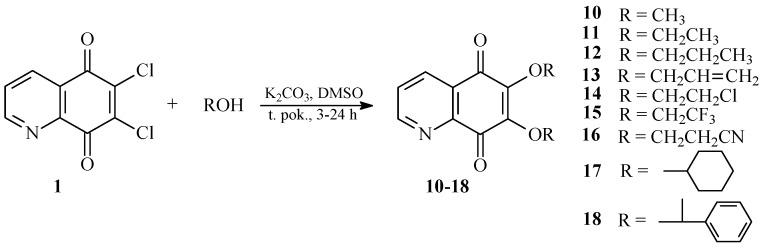
Synthesis of the 6,7-disubstituted derivatives of 5,8-quinolinedione, **10**–**18**.

In our work, dimethyl sulfoxide (DMSO) was used as a solvent in order to obtain 6,7-dialkoxy derivatives. Treatment of 6,7-dichloro-5,8-quinolinedione **1** with alcohols in DMSO in the presence of potassium carbonate gave 6,7-disubstituted products **10**–**18** with the good yields (Scheme 2).

Structures of all new compounds **10**–**18** were confirmed by ^1^H-, ^13^C-NMR, IR and MS spectra. In addition, the crystal structure of the derivative **15** was determined by the X-ray diffraction analysis.

**Scheme 3:**
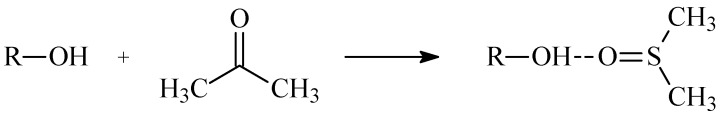
Forming the hydrogen bond between alcohol molecule and DMSO.

Formation of the disubstituted products in this reaction can be explained by the influence of the solvent on the alcohol nucleophilicity. In the presence of the dimethyl sulfoxide, the DMSO-alcohol complex can be formed via the hydrogen bond (Scheme 3) [20,21].

Formation of the H-bond leads to an increase in the nucleophilic character of the alcohol molecule, enabling the substitution of two chlorine atoms into the 5,8-quinolinedione moiety. In tetrahydrofuran, the interaction between solvent and alcohol molecule does not occur, giving the monoalkoxy derivative as the product of the reaction.

### 2.2. Crystal Structure

**Figure 2:**
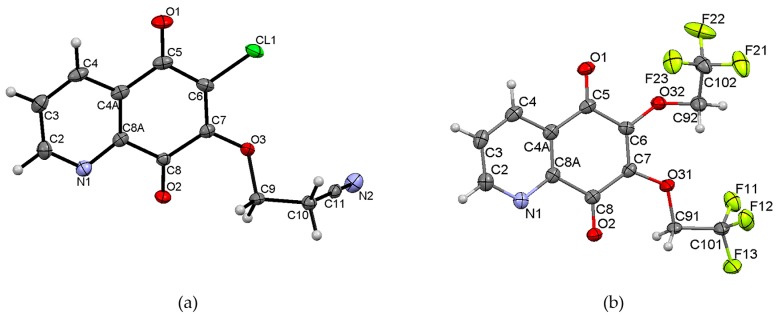
Molecular structures with the atom numbering of (**a**) 6-chloro-7-(2-cyanoethoxy)-5,8-quinolinedione **7** and (**b**) 6,7-di(2,2,2-trifluoroethoxy)-5,8-quinolinedione **15**. Displacement ellipsoids are shown at the 50% probability level. H atoms are shown as small spheres of arbitrary radii.

X-ray diffraction analysis was performed for monocrystals of the following compounds: 6-chloro-7-cyanoethoxy-5,8-quinolinedione **7** and 6,7-di(2,2,2-trifluoroethoxy)-5,8-quinolinedione **15**. Both crystals were grown from dichloromethane. Figure 2 presents the molecular structure and atom numbering of the crystals. The derivatives **7** and **15** were crystallized in different monoclinic space groups, C 2/c and *P*2_1_/n, respectively.

**Figure 3:**
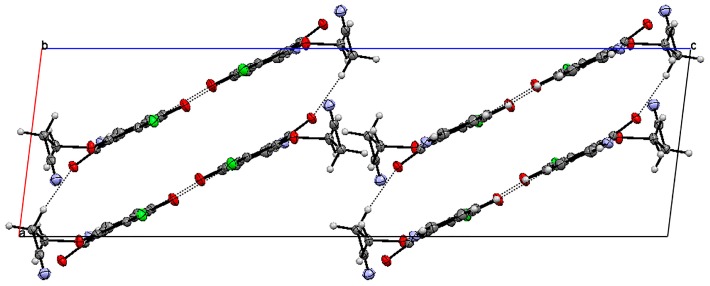
Molecular arrangement in 6-chloro-7-(2-cyanoethoxy)-5,8-quinolinedione **7** showing a “ring” pattern. View down the “b” axis.

The unit cell of **7** contains eight molecules (Table S1). The angle between mean planes of rings and the 2-cyanoethoxy chain O3-C9-C10 is equal to 33.36°, while a similar angle in the crystal structures of 6-(7-chloro-6-(4-methoxyphenyl)-5,8-quinolinedione [15], 6-chloro-7-propylamine -5,8-quinolinedione, and 7-chloro-6-propylamine-5,8-quinolinedione [17] were equal to 82.03°, 89.77°, and 81.79°, respectively. This significant difference results from the various molecular packing that occurs in these crystals. As shown in Figure 3, molecules of **7** are connected by weak hydrogen bonds, which form the “ring” pattern. The 5,8-quinolinedione moiety is located inside the ring, whereas the substituent chain is arranged outside of the ring. All parameters of the hydrogen bonds are shown in Table S3.

Moreover, the neighboring rings are linked by the following hydrogen bonds: C9-H9B···N2 and C10-H10B···N1 (Figure S1, Table S3). Both interactions have a highly bent geometry, which does not really correspond to a significant hydrogen bond interaction. According to the literature data [17,22], the hydrogen bonds character decreases with a decreasing <DHA angle.

**Figure 4:**
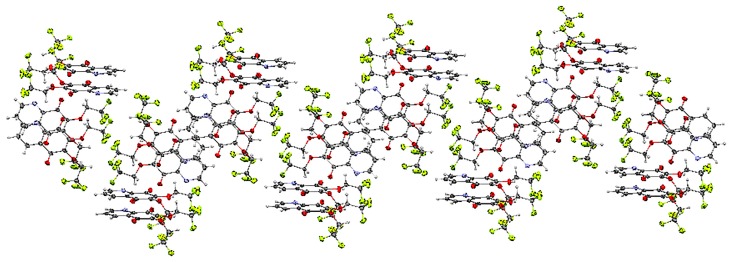
Molecular arrangement in the 6,7-(2,2,2-trifloroethoxy)-5,8-quinolinedione **15** crystal showing a “spiral” pattern. View down the “b” axis.

The unit cell of **15** contains four molecules (Table S1). The molecules form an infinite spiral along the “b” axis, as shown in Figure 4. The angles between the mean planes of rings and the 2,2,2-trifluoroethoxy chain at positions C-7 (O31C91C101) and C-6 (O32C92C102) are equal to 14.37° and 56.16°, respectively.

The crystal structure of 6,7-di(2,2,2-trifluoro)-5,8-quinolinedione **15** is stabilized by the intra- and inter-unit hydrogen bonds, which are formed between the C-H groups as H-bond donors (D) and N, O or fluorine atoms as H-bond acceptors (A) (Table S3). The C-H···O, C-H···N and C-H···F hydrogen bonds are moderately strong, as their H···A distances cover the range of 2.35–2.64 Å (Figure S2) [22].

### 2.3. Cytotoxic Activity

The antiproliferative activities *in vitro* of the new compounds **2**–**18** and for 6,7-dichloro-5,8-quinolinedione **1** were tested against the three human cancer lines: melanoma (C-32), glioblastoma (SNB-19), and breast cancer (MDA-MB-231), as well as normal fibroblasts cell lines (HFF-1) using the WST-1 test. The IC_50_ parameter (µM), defined as the compound concentration that inhibits the proliferation of 50% of tumor cells as compared to the control untreated cells, was used to characterize all cytotoxic activities under study. As a reference, the cytotoxic agent cisplatin was applied. The results of the cytotoxicity analysis are collected in Table 1.

As shown in Table 1, the introduction of the alkoxy group at the C7 and/or C6 position leads to changes in the antiproliferative activity against the tested cancer lines compared to 6,7-dichloro-5,8-quinolinedione **1**. The series of the 7-subtsituted derivatives **2**–**9**, except for compound **7**, exhibits a high activity against melanoma (C-32) cancer cell lines. Moreover, compounds **4**–**5** and **8**–**9** show higher activity than cisplatin. Compound **9** exhibits a selective effect for the C-32 lines with the IC_50_ value being twenty-five and twenty times higher than those for 6,7-dichloro-5,8-quinolinedione **1** and cisplatin, respectively. In the series of monosubstituted derivatives **2**–**9**, 6-chloro-7-methoxy-5,8-quinolinedione **2** shows the highest cytotoxic activity against the human breast cancer (MDA-MB-231) cell line and its selectivity index is equal to 18.4. Compound **2** is nearly seventeen times more active than both compound **1** and cisplatin. The introduction of the cyanoethoxy or chloroethoxy substituents to the 5,8-quinolinedione moiety in compounds **5** and **6** leads to a decrease in the cytotoxic activity. The presence of a double bond in the substituent causes an increase in the activity against melanoma cells (C-32). The structure–activity relationship indicates that the rank order of the cytotoxic activity against the glioblastoma (SNB-19) cell line observed in the 7-substituted derivatives **2**–**5** is as follows: propoxy > ethoxy > methoxy; *i.e.*, it is influenced by the length of the chain substituent.

Comparing the activity of the mono- and disubtituted derivatives, one can notice that the introduction of the two alkoxy groups at the C6 and C7 positions leads to changes in the anticancer activity against tested human cancer lines. For the derivatives **10**–**18**, only compounds **11** and **14** show a higher activity than the monosubstituted derivatives **2**–**9** with respect to the C-32 cancer line. Derivatives **8** and **17** exhibit very similar cytotoxic effects against both the melanoma (C-32) and glioblastoma (SNB-19) cell lines. Thus, the replacement of the chlorine atom in the cyclohexyloxy group did not change the activity of the compound. Moreover, compound **15** exhibits a selectivity effect against breast cancer (MDA–MB-231) cell line, for which the IC_50_ value is equal to 2.7 ± 0.1 µM. The selectivity index (SI = 4.6) is twenty times higher than that of cisplatin. For derivatives **11**–**16**, the rank order of the anticancer activity against the SNB-19 cell lines is as follows: **13** > **12** > **11** > **10** = **14** > **15** > **16**. Similar to the monosubstituted derivatives, the substituent chain length and the double bond lead to an increase in the cytotoxic activity. On the other hand, the halogen atoms and cyano group causes a reduction in the cytotoxicity.

**Table 1:** Cytotoxic activity of 6,7-dichloro-5,8-quinolinedione **1**, alkoxy derivatives of 5,8-quinolinedione **2**–**18** and cisplatin as a reference compound. The incubation time is 72 h.

Compound	Human Cell Line/IC_50_ ± SD [µM]				
	R	C-32	SNB-19	MDA-MB-231	HFF-1
**1**	-	22.8 ± 0.7	26.2 ± 0.8	25.1 ± 3.1	15.7 ± 0.5
**2**	CH_3_	19.2 ± 2.1	42.2 ± 0.1	1.5 ± 0.2	27.6 ± 2.1
**3**	CH_2_CH_3_	30.7 ± 1.1	10.3 ± 2.4	24.0 ± 2.8	1.3 ± 0.5
**4**	CH_2_CH_2_CH_3_	0.20 ± 0.01	0.36 ± 0.03	22.4 ± 2.7	Neg
**5**	CH_2_CH =CH_2_	0.28 ± 0.09	0.30 ± 0.01	17.2 ± 2.8	Neg
**6**	CH_2_CH_2_Cl	22.8 ± 2.5	18.3 ± 1.4	17.0 ± 3.0	15.8 ± 1.2
**7**	CH_2_CH_2_CN	119.4 ± 4.3	58.3 ± 2.8	164.5 ± 3.9	2.5 ± 0.4
**8**	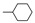	1.4 ± 0.1	1.8 ± 0.1	3.5 ± 0.3	13.9 ± 3.0
**9**	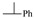	0.81 ± 0.01	2.3 ± 0.5	271.3 ± 9.8	2.0 ± 0.3
**10**	CH_3_	21.5 ± 2.0	3.5 ± 0.7	3.1 ± 0.1	3.1 ± 0.8
**11**	CH_2_CH_3_	17.6 ± 1.6	2.1 ± 0.1	18.6 ± 2.0	Neg
**12**	CH_2_CH_2_CH_3_	1.1 ± 0.1	0.7 ± 0.02	3.0 ± 0.3	Neg
**13**	CH_2_CH =CH_2_	1.5 ± 0.01	0.2 ± 0.01	12.6 ± 2.7	Neg
**14**	CH_2_CH_2_Cl	12.1 ± 0.2	3.5 ± 0.3	10.9 ± 1.4	1.9 ± 0.2
**15**	CH_2_CH_3_	10.9 ± 1.3	14.9 ± 0.7	2.7 ± 0.1	12.4 ± 1.2
**16**	CH_2_CH_2_CN	134.4 ± 10.7	203.7 ± 14.3	110.7 ± 3.7	18.2 ± 2.6
**17**	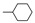	1.4 ± 0.1	1.7 ± 0.3	13.8 ± 0.1	Neg
**18**	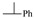	18.7 ± 0.9	137.4 ± 0.7	107.9 ± 10.5	17.2 ± 1.7
cisplatin		16.4 ± 1.2	18.9 ± 0.4	25.5 ± 0.2	9.1 ± 1.8

Neg—Negative in the concentration used.

## 3. Materials and Methods

### 3.1. General Techniques

Melting points were measured on an Electrothermal IA 9300 melting point apparatus. The ^1^H- and ^13^C-NMR spectra were determined using a Bruker Avance 600 spectrometer (Bruker, Billerica, MA, USA) in CDCl_3_; chemical shifts (δ) are reported in ppm and *J* values in Hz. The peak multiplicity is designated as singlet (s), doublet (d), triplet (t), doublet of doublets (dd), doublet of triplets (dt) and multiplet (m). Mass spectra were recorded under EI conditions on a Finnigan MAT 95 instrument (Thermo Fisher Scientific, Waltham, MA, USA). High-resolution mass spectral analysis was performed on a Bruker Impact II instrument (Bruker). Infrared spectra were recorded on a IRAffinity-1 Shimadzu spectrophotometer (Shimadzu Corporation, Kioto, Japan). Thin layer chromatography (TLC) was carried out on silica gel 60 254F plates (Merck, Darmstadt, Germany) using a mixture of chloroform and ethanol (40:1 or 15:1, *v*/*v*) as an eluent. The spots were visualized by UV light (254 nm) and iodine. All new compounds were purified by column chromatography. As a solid phase silica gel 60 was used and the eluent was a mixture of chloroform and ethanol (40:1, *v*/*v*).

The starting compound, 6,7-dichloro-5,8-quinolinedione **1**, was obtained according to methods described previously [16,17]. M.p. 220–221 °C (lit. m.p. 219–221 °C [16]; 220–221 °C [17]).

### 3.2. General Procedure for the Synthesis of 6-Chloro-7-alkoxy-5,8-quinolinedione

The mixture of 6,7-dichloro-5,8-quinolinedione **1** (0.100 g, 0.441 mmol) and potassium carbonate (0.061 g, 0.441 mmol) in dry tetrahydrofuran (1 mL) was added to a solution of alcohol (1.2 eqv., 0.529 mmol) in dry tetrahydrofuran (0.5 mL). Stirring at room temperature was continued for 3–24 h. Subsequently, the reaction mixture was concentrated under reduced pressure. The crude product was purified by column chromatography (chloroform/ethanol, 40:1, *v*/*v*) to give pure product **2**–**9**.

*6-Chloro-7-methoxy-5,8-quinolinedione* (**2**). Yield: 53%, m.p. 161–163 °C. ^1^H-NMR (CDCl_3_, 600 MHz) δ 4.42 (s, 3H, OCH_3_), 7.72 (dd, *J*_23_ = 4.8 Hz, *J*_34_ = 7.8 Hz, 1H, H-3), 8.50 (dd, *J*_24_ = 1.8 Hz, *J*_34_ = 7.8 Hz, 1H, H-4), 9.06 (dd, *J*_24_ = 1.8 Hz, *J*_23_ = 4.8 Hz, 1H, H-2). ^13^C-NMR (CDCl_3_, 150 MHz) δ 62.2 (OCH_3_), 127.6 (C-6), 128.0 (C-3), 128.1 (C-4a), 134.9 (C-4), 146.6 (C-8a), 154.7 (C-2), 157.4 (C-7), 177.7 (C-8), 177.9 (C-5). EI MS (70 eV) *m*/*z*: 223 [M^+^] (100), 193 (31), 152 (9), 137 (36), 124 (21), 89 (2), 77 (8). IR (KBr, cm^−1^) ν_max_: 3078–2857, 1696, 1662, 1594–1560, 1096. HR-MS (APCI) *m*/*z*: C_10_H_6_ClNO_3_ [M + H]^+^, Calcd. 224.0114; Found 224.0101.

*6-Chloro-7-ethoxy-5,8-quinolinedione* (**3**). Yield: 58%, m.p. 112–114 °C. ^1^H-NMR (CDCl_3_, 600 MHz) δ 1.49 (t, *J* = 7.8 Hz, 3H, CH_3_), 4.73 (q, *J* = 6.6 Hz, *J* = 7.8 Hz, 2H, OCH_2_), 7.69 (dd, *J*_23_ = 4.8 Hz, *J*_34_ = 7.8 Hz, 1H, H-3), 8.47 (dd, *J*_24_ = 1.8 Hz, *J*_34_ = 7.8 Hz, 1H, H-4), 9.03 (dd, *J*_24_ = 1.8 Hz, *J*_23_ = 4.8 Hz, 1H, H-2). ^13^C-NMR (CDCl_3_, 150 MHz) δ 16.0 (CH_2_CH_3_), 71.2 (OCH_2_), 127.9 (C-6), 128.2 (C-3), 128.5 (C-4a), 134.8 (C-4), 146.6 (C-8a), 154.7 (C-2), 157.3 (C-7), 177.8 (C-8), 178.0 (C-5). EI MS (70 eV) *m*/*z*: 237 [M^+^] (100), 222 (12), 193 (67), 181 (65), 137 (98), 90 (10), 77 (10). IR (KBr, cm^−1^) ν_max_: 3080–2850, 1691, 1669, 1591–1560, 1094. HR-MS (APCI) *m*/*z*: C_11_H_8_ClNO_3_ [M + H]^+^, Calcd. 238.0271; Found 238.0261.

*6-Chloro-7-propoxy-5,8-quinolinedione* (**4**). Yield: 87%, m.p. 121–122 °C. ^1^H-NMR (CDCl_3_, 600 MHz) δ 1.07 (t, *J* = 7.2 Hz, 3H, CH_3_), 1.85–1.87 (m, 2H, CH_2_CH_3_), 4.63 (t, *J* = 6.6 Hz, OCH_2_), 7.70 (dd, *J*_23_ = 4.8 Hz, *J*_34_ =7.8 Hz, 1H, H-3), 8.48 (dd, *J*_24_ = 1.8 Hz, *J*_34_ = 7.8 Hz, 1H, H-4), 9.05 (dd, *J*_24_ = 1.8 Hz, *J_23_* = 4.8 Hz, 1H, H-2). ^13^C-NMR (CDCl_3_, 150 MHz) δ 10.2 (CH_3_), 23.8 (CH_2_CH_3_), 75.8 (OCH_2_), 127.9 (C-6), 128.1 (C-3), 128.1 (C-4a), 134.8 (C-4), 146.6 (C-8a), 154.6 (C-2), 157.4 (C-7), 177.7 (C-8), 178.0 (C-5). EI MS (70 eV) *m*/*z*: 251 [M^+^] (46), 209 (50), 181 (100), 137 (75), 125 (15), 90 (8), 70 (11). IR (KBr, cm^−1^) ν_max_: 3042–2898, 1694, 1671, 1596–1570, 1097. HR-MS (APCI) *m*/*z*: C_12_H_10_ClNO_3_ [M + H]^+^, Calcd. 252.0427; Found 252.0417.

*6-Chloro-7-(2-propenoxy)-5,8-quinolinedione* (**5**). Yield: 88%, m.p. 89–90 °C. ^1^H-NMR (CDCl_3_, 600 MHz) δ 5.19 (dt, *J* = 1.2 Hz, *J* = 6.0 Hz, 2H, OCH_2_), 5.33 (dt, *J* = 1.2 Hz, *J* = 10.2 Hz, 1H, CH=CH_2_), 5.48 (dt, *J* = 1.2 Hz, *J* = 16.2 Hz, 1H, CH=CH_2_), 6.04–6.10 (m, 1H, CH=CH_2_), 7.69 (dd, *J*_23_ = 4.8 Hz, *J*_34_ = 7.8 Hz, 1H, H-3), 8.47 (dd, *J*_24_ = 1.8 Hz, *J*_34_ = 7.8 Hz, 1H, H-4), 9.03 (dd, *J*_24_ = 1.8 Hz, *J*_23_ = 4.8 Hz, 1H, H-2). ^13^C-NMR (CDCl_3_, 150 MHz) δ 75.0 (OCH_2_), 120.0 (CH=CH_2_), 127.9 (C-6), 128.1 (C-3), 128.2 (C-4a), 132.4 (CH=CH_2_), 134.9 (C-4), 146.6 (C-8a), 154.7 (C-2), 156.8 (C-7), 177.7 (C-8), 178.0 (C-5). EI MS (70 eV) *m*/*z*: 249 [M^+^] (10), 221 (59), 193 (6), 176 (53), 124 (33), 90 (6), 77 (23). IR (KBr, cm^−1^) ν_max_: 3038, 1694, 1671, 1595–1569, 1097. HR-MS (APCI) *m*/*z*: C_12_H_8_ClNO_3_ [M + H]^+^, Calcd. 250.0271; Found 250.0265.

*6-Chloro-7-(2-chloroethoxy)-5,8-quinolinedione* (**6**). Yield: 78%, m.p. 114–116 °C. ^1^H-NMR (CDCl_3_, 600 MHz) δ 3.86 (t, *J* = 6.0 Hz, 2H, CH_2_Cl), 4.89 (t, *J* = 0.6 Hz, 2H OCH_2_), 7.72 (dd, *J*_23_ = 4.8 Hz, *J*_34_ = 7.8 Hz, 1H, H-3), 8.49 (dd, *J*_24_ = 1.8 Hz, *J*_34_ = 7.8 Hz, 1H, H-4), 9.05 (dd, *J*_24_ = 1.8 Hz, *J*_23_ = 4.8 Hz, 1H, H-2). ^13^C-NMR (CDCl_3_, 150 MHz) δ 42.9 (CH_2_Cl), 74.0 (OCH_2_), 127.7 (C-6), 128.1 (C-3), 129.2 (C-4a), 134.9 (C-4), 146.5 (C-8a), 154.8 (C-2), 156.7 (C-7), 177.6 (C-8), 177.8 (C-5). EI MS (70 eV) *m*/*z*: 273 [M^+^] (25), 236 (100), 209 (31), 181 (66), 137 (89), 90 (15), 77 (33). IR (KBr, cm^−1^) ν_max_: 3078–2958, 1685, 1668, 1593–1564, 1096. HR-MS (APCI) *m*/*z*: C_11_H_7_Cl_2_NO_3_ [M + H]^+^, Calcd. 271.9881; Found 271.9872.

*6-Chloro-7-(2-cyanoethoxy)-5,8-quinolinedione* (**7**): Yield: 68%, m.p. 177–179 °C. ^1^H-NMR (CDCl_3_, 600 MHz) δ 3.02 (t, *J* = 5.4 Hz, 2H, CH_2_CN), 4.71 (t, *J* = 6.0 Hz, 2H, OCH_2_), 7.85 (dd, *J*_23_ = 4.8 Hz, *J*_34_ = 7.8 Hz, 1H, H-3), 8.40 (dd, *J*_24_ = 1.8 Hz, *J*_34_ = 7.8 Hz, 1H, H-4), 9.02 (dd, *J*_24_ = 1.8 Hz, *J*_23_ = 4.8 Hz, 1H, H-2). ^13^C-NMR (CDCl_3_, 150 MHz) δ 19.0 (CH_2_CN), 68.7 (OCH_2_), 118.4 (CH_2_CN), 127.8 (C-6), 128.0 (C-3, C-4a), 134.1 (C-4), 146.9 (C-8a), 154.2 (C-2), 156.9 (C-7), 177.1 (C-8), 177.9 (C-5). EI MS (70 eV) *m*/*z*: 262 [M^+^] (38), 227 (20), 222 (56), 193 (19), 137 (100), 90 (17), 77 (34). IR (KBr, cm^−1^) ν_max_: 3079, 2965, 2917, 2257, 1691, 1666, 1596–1568, 1094. HR-MS (APCI) *m*/*z*: C_12_H_7_ClN_2_O_3_ [M + H]^+^, Calcd. 263.0223; Found 263.0219.

*6-Chloro-7-cyclohexyloxy-5,8-quinolinedione* (**8**): Yield: 79%, m.p. 123–124 °C. ^1^H-NMR (CDCl_3_, 600 MHz) δ 1.37–1.40 (m, 4H, 2 × CH_2_), 1.69–1.72 (m, 2H, CH_2_), 1.82–1.84 (m, 2H, CH_2_), 1.97–1.99 (m, 2H, CH_2_), 5.17–5.20 (m, 1H, OCH), 7.69 (dd, *J*_23_ = 4.8 Hz, *J*_34_ = 7.8 Hz, 1H, H-3), 8.48 (dd, *J*_24_ = 1.8 Hz, *J*_34_ = 7.8 Hz, 1H, H-4), 9.02 (dd, *J*_24_ = 1.8 Hz, *J*_23_ = 4.8 Hz, 1H, H-2). ^13^C-NMR (CDCl_3_, 150 MHz) δ 23.3 (CH_2_), 25.2 (2 × CH_2_), 32.8 (2 × CH_2_), 83.2 (OCH_2_), 127.8 (C-3), 128.2 (C-6), 129.9 (C-4a), 134.8 (C-4), 146.7 (C-8a), 154.6 (C-2), 157.2 (C-7), 177.8 (C-8), 178.2 (C-5). EI MS (70 eV) *m*/*z*: 291 [M^+^] (8), 211 (92), 181 (65), 137 (42), 90 (8), 77 (15), 55 (100). IR (KBr, cm^−1^) ν_max_: 3048, 2948–2854, 1682, 1665, 1594–1565, 1096. HR-MS (APCI) *m*/*z*: C_15_H_14_ClNO_3_ [M + H]^+^, Calcd. 292.0740; Found 292.0730.

*6-Chloro-7-(1-methylbenzyloxy)-5,8-quinolinedione* (**9**): Yield: 65%, m.p. 215–217 °C. ^1^H-NMR (CDCl_3_, 600 MHz) δ 1.77 (d, *J* = 6.6 Hz, 3H, CH_3_), 6.49 (q, *J* = 3.6 Hz, *J* = 6.6 Hz, 1H, CHCH_3_), 7.25–7.26 (m, 1H, Ph), 7.30–7.31 (m, 2H, Ph), 7.42–7.43 (m, 2H, Ph), 7.64 (dd, *J*_23_ = 4.8 Hz, *J*_34_ = 7.8 Hz, 1H, H-3), 8.39 (dd, *J*_24_ = 1.8 Hz, *J*_34_ = 7.8 Hz, 1H, H-4), 8.97 (dd, *J*_24_ = 1.8 Hz, *J*_23_ = 4.8 Hz, 1H, H-2). ^13^C-NMR (CDCl_3_, 150 MHz) δ 23.9 (CH_3_), 62.6 (CH), 126.5 (Ph), 127.8 (C-3), 128.0 (Ph), 128.3 (C-6), 128.4 (Ph), 128.6 (Ph), 128.7 (Ph), 130.2 (C-4a), 134.8 (C-4), 141.1 (Ph), 146.5 (C-8a), 154.6 (C-2), 156.7 (C-7), 177.6 (C-8), 178.2 (C-5). EI MS (70 eV) *m*/*z*: 313 [M^+^] (9), 278 (21), 211 (20), 181 (15), 137 (10), 105 (100), 77 (30). IR (KBr, cm^−1^) ν_max_: 3064–2854, 1690, 1671, 1592–1559, 1094. HR-MS (APCI) *m/z*: C_17_H_12_ClNO_3_ [M + H]^+^, Calcd. 314.0584; Found 314.0572.

### 3.3. General Procedure for the Synthesis of 6,7-Dialkoxy-5,8-quinolinedione

The mixture of 6,7-dichloro-5,8-quinolinedione **1** (0.100 g, 0.441 mmol) and potassium carbonate (0.138 g, 0.882 mmol) in dry dimethyl sulfoxide (1 ml) was added to a solution of alcohol (2.2 eqv., 0.970 mmol) in dry dimethyl sulfoxide (0.5 mL). Stirring at room temperature was continued for 3–24 h. Subsequently, the reaction mixture was concentrated under reduced pressure. The crude product was purified by column chromatography (chloroform/ethanol, 40:1, *v*/*v*) to give pure product **10**–**18**.

*6,7-Dimethoxy-5,8-quinolinedione* (**10**): Yield: 49%, m.p. 132–133 °C. ^1^H-NMR (CDCl_3_, 600 MHz) δ 4.17 (s, 3H, CH_3_), 4.19 (s, 3H, CH_3_), 7.67 (dd, *J_23_* = 4.8 Hz, *J*_34_ = 7.8 Hz, 1H, H-3), 8.43 (dd, *J*_24_ = 1.8 Hz, *J*_34_ = 7.8 Hz, 1H, H-4), 9.02 (dd, *J*_24_ = 1.8 Hz, *J*_23_ = 4.8 Hz, 1H, H-2). ^13^C-NMR (CDCl_3_, 150 MHz) δ 61.6 (OCH_3_), 61.7 (OCH_3_), 127.5 (C-3), 127.7 (C-4a), 134.3 (C-4), 146.7 (C-8a), 147.2 (C-7), 148.4 (C-6), 154.5 (C-2), 180.2 (C-8), 180.9 (C-5). EI MS (70 eV) *m*/*z*: 221 [M^+^] (9), 204 (100), 189 (69), 174 (66), 148 (37), 105 (71), 77 (63). IR (KBr, cm^−1^) ν_max_: 3024–2845, 1690, 1672, 1607–1570. HR-MS (APCI) *m*/*z*: C_11_H_9_NO_4_ [M + H]^+^, Calcd. 220.0609; Found 220.0600.

*6,7-Diethoxy-5,8-quinolinedione* (**11**): Yield: 65%, m.p. 69–70 °C. ^1^H-NMR (CDCl_3_, 600 MHz) δ 1.46 (t, *J* = 13.8 Hz, 6H, 2 × CH_3_), 4.41 (m, 4H, 2 × OCH_2_), 7.65 (dd, *J*_23_ = 4.8 Hz, *J*_34_ = 7.8 Hz, 1H, H-3), 8.42 (dd, *J*_24_ = 1.8 Hz, *J*_34_ = 7.8 Hz, 1H, H-4), 9.01 (dd, *J*_24_ = 1.8 Hz, *J*_23_ = 4.8 Hz, 1H, H-2). ^13^C-NMR (CDCl_3_, 150 MHz) δ 15.7 (2 × CH_3_), 70.0 (OCH_2_), 127.4 (C-3), 127.7 (C-4a), 134.2 (C-4), 146.8 (C-7), 147.1 (C-6), 148.2 (C-8a), 154.4 (C-2), 180.5 (C-8), 181.2 (C-5). EI MS (70 eV) *m*/*z*: 247 [M^+^] (13), 232 (65), 218 (100), 203 (54), 192 (54), 106 (26), 79 (72). IR (KBr, cm^−1^) ν_max_: 3078–2904, 1684, 1669, 1602–1560. HR-MS (APCI) *m/z*: C_13_H_13_NO_4_ [M + H]^+^, Calcd. 248.0922; Found 248.0912.

*6,7-Dipropoxy-5,8-quinolinedione* (**12**): Yield: 62%, m.p. 133–135 °C. ^1^H-NMR (CDCl_3_, 600 MHz) δ 1.03–1.05 (m, 6H, 2 × CH_3_), 1.81–1.86 (m, 4H, 2 × CH_2_CH_3_), 4.30 (t, *J* = 6.6 Hz, 2H, OCH_2_), 4.33 (t, *J* = 6.6 Hz, 2H, OCH_2_), 7.63 (dd, *J*_23_ = 4.8 Hz, *J*_34_ = 7.8 Hz, 1H, H-3), 8.38 (dd, *J*_24_ = 1.8 Hz, *J*_34_ = 7.8 Hz, 1H, H-4), 8.98 (dd, *J*_24_ = 1.8 Hz, *J*_23_ = 4.8 Hz, 1H, H-2). ^13^C-NMR (CDCl_3_, 150 MHz) δ 10.2 (2 × CH_3_), 23.5 (2 × CH_2_CH_3_), 75.8 (OCH_2_), 75.9 (OCH_2_), 127.3 (C-3), 127.7 (C-4a), 134.1 (C-4), 146.9 (C-7), 147.4 (C-6), 148.8 (C-8a), 154.3 (C-2), 180.4 (C-8), 181.1 (C-5). EI MS (70 eV) *m*/*z*: 275 [M^+^] (9), 246 (27), 233 (33), 191 (93), 163 (100), 135 (50), 79 (40). IR (KBr, cm^−1^) ν_max_: 2968–2879, 1671, 1604, 1579–1570. HR-MS (APCI) *m*/*z*: C_15_H_17_NO_4_ [M + H]^+^, Calcd. 276.1235; Found 276.1225.

*6,7-di(2-Propenyloxy)-5,8-quinolinedione* (**13**): Yield: 71%, m.p. 135–136 °C. ^1^H-NMR (CDCl_3_, 600 MHz) δ 4.90 (dt, *J* = 1.2 Hz, *J* = 6.0 Hz, 2H, OCH_2_), 4.93 (dt, *J* = 1.2 Hz, *J* = 6.0 Hz, 2H, OCH_2_), 5.30 (dt, *J* = 1.2 Hz, *J* = 9.0 Hz, 2H, 2 × CH=CH_2_), 5.43 (dt, *J* = 1.2 Hz, *J* = 16.2 Hz, 2H, 2 × CH=CH_2_), 6.04–6.10 (m, 2H, 2 × CH=CH_2_), 7.64 (dd, *J*_23_ = 4.8 Hz, *J*_34_ = 7.8 Hz, 1H, H-3), 8.40 (dd, *J*_24_ = 1.8 Hz, *J*_34_ = 7.8 Hz, 1H, H-4), 8.99 (dd, *J*_24_ = 1.8 Hz, *J*_23_ = 4.8 Hz, 1H, H-2). ^13^C-NMR (CDCl_3_, 150 MHz) δ 74.5 (2 × OCH_2_), 119.4 (CH=CH_2_), 119.6 (CH=CH_2_), 127.5 (C-3), 127.7 (C-4a), 132.8 (2 × CH=CH_2_), 134.3 (C-4), 146.8 (C-6), 147.1 (C-7), 148.3 (C-8a), 154.5 (C-2), 180.3 (C-8), 181.0 (C-5). EI MS (70 eV) *m*/*z*: 271 [M^+^] (1), 230 (59), 213 (19), 174 (22), 146 (17), 105 (19), 77 (31). IR (KBr, cm^−1^) ν_max_: 3075–2929, 1696, 1676, 1648–1517. HR-MS (APCI) *m*/*z*: C_15_H_13_NO_4_ [M + H]^+^, Calcd. 272.0922; Found 272.0910.

*6,7-di(2-Chloroethoxy)-5,8-quinolinedione* (**14**): Yield: 65%, m.p. 189–191 °C. ^1^H-NMR (CDCl_3_, 600 MHz) δ 3.85–3.88 (m, 4H, 2 × CH_2_Cl), 4.67–4.69 (m, 4H, 2 × OCH_2_), 7.66 (dd, *J*_23_ = 4.8 Hz, *J*_34_ = 7.8 Hz, 1H, H-3), 8.40 (dd, *J*_24_ = 1.8 Hz, *J*_34_ = 7.8 Hz, 1H, H-4), 9.00 (dd, *J*_24_ = 1.8 Hz, *J*_23_ = 4.8 Hz, 1H, H-2). ^13^C-NMR (CDCl_3_, 150 MHz) δ 42.7 (CH_2_Cl), 42.8 (CH_2_Cl), 73.5 (OCH_2_), 73.5 (OCH_2_), 127.6 (C-3), 127.6 (C-4a), 134.3 (C-4), 146.6 (C-7), 147.7 (C-6), 147.8 (C-8a), 154.6 (C-2), 179.9 (C-8), 180.5 (C-5). EI MS (70 eV) *m*/*z*: 317 [M^+^] (11), 280 (76), 218 (100), 192 (45), 164 (25), 90 (14), 77 (57). IR (KBr, cm^−1^) ν_max_: 3457, 1804, 1776, 1667–1610, 1077. HR-MS (APCI) *m*/*z*: C_13_H_11_Cl_2_NO_4_ [M + H]^+^, Calcd. 316.0143; Found 316.0133.

*6,7-di(2,2,2-Trifluoroethoxy)-5,8-quinolinedione* (**15**): Yield: 82%, m.p. 110–111 °C. ^1^H-NMR (CDCl_3_, 600 MHz) δ 4.79 (q, *J* = 8.4 Hz, *J* = 7.8 Hz, 2H, OCH_2_), 4.85 (q, *J* = 8.4 Hz, *J* = 7.8 Hz, 2H, OCH_2_), 7.71 (dd, *J_23_* = 4.8 Hz, *J*_34_ = 7.8 Hz, 1H, H-3), 8.42 (dd, *J*_24_ = 1.8 Hz, *J*_34_ = 7.8 Hz, 1H, H-4), 9.05 (dd, *J*_24_ = 1.8 Hz, *J*_23_ = 4.8 Hz, 1H, H-2). ^13^C-NMR (CDCl_3_, 150 MHz) δ 69.1–69.6 (CH_2_), 127.4 (CF), 128.0 (C-3), 129.7 (C-4a), 134.5 (C-4), 145.7 (C-7), 146.2 (C-6), 146.7 (C-8a), 155.0 (C-2), 179.1 (C-8), 179.7 (C-5). EI MS (70 eV) *m*/*z*: 355 [M^+^] (33), 336 (18), 286 (61), 272 (100), 174 (47), 105 (75), 77 (50). IR (KBr, cm^−1^) ν_max_: 2959–2855, 1685, 1667, 1596–1560, 1101-1066. HR-MS (APCI) *m*/*z*: C_13_H_7_F_6_NO_4_ [M + H]^+^, Calcd. 356.0357; Found 356.0341.

*6,7-di(2-Cyanoethoxy)-5,8-quinolinedione* (**16**): Yield: 59%, m.p. 45–47 °C. ^1^H-NMR (CDCl_3_, 600 MHz) δ 2.95–2.98 (m, 4H, 2 × CH_2_CN), 4.66–4.68 (m, 4H, 2 × OCH_2_), 7.71 (dd, *J*_23_ = 4.8 Hz, *J*_34_ = 7.8 Hz, 1H, H-3), 8.43 (dd, *J*_24_ = 1.8 Hz, *J*_34_ = 7.8 Hz, 1H, H-4), 9.05 (dd, *J*_24_ = 1.8 Hz, *J*_23_ = 4.8 Hz, 1H, H-2). ^13^C-NMR (CDCl_3_, 150 MHz) δ 18.6 (2 × CH_2_), 67.3 (2 × OCH_2_), 116.1 (CN), 116.2 (CN), 126.5 (C-3), 126.9 (C-4a), 133.4 (C-4), 145.4 (C-7), 145.6 (C-6), 146.6 (C-8a), 153.8 (C-2), 178.8 (C-8), 179.3 (C-5). EI MS (70 eV) *m*/*z*: 297 [M^+^] (19), 257 (41), 243 (50), 215 (89), 192 (100), 105 (42), 78 (62). IR (KBr, cm^−1^) ν_max_: 2966–2850, 2253, 1669, 1611–1581. HR-MS (APCI) *m*/*z*: C_15_H_11_N_3_O_4_ [M + H]^+^, Calcd. 298.0827; Found 298.0817.

*6,7-Dicyclohexloxy-5,8-quinolinedione* (**17**): Yield: 51%, oil. ^1^H-NMR (CDCl_3_, 600 MHz) δ 1.26–1.36 (m, 8H, 4 × CH_2_), 1.54–1.58 (m, 4H, 2 × CH_2_), 1.71–1.74 (m, 4H, 2 × CH_2_), 1.98–2.04 (m, 4H, 2 × CH_2_), 4.69–4.70 (m, 1H, OCH), 4.77–4.78 (m, 1H, OCH), 7.61 (dd, *J*_23_ = 4.8 Hz, *J*_34_ = 7.8 Hz, 1H, H-3), 8.37 (dd, *J*_24_ = 1.8 Hz, *J*_34_ = 7.8 Hz, 1H, H-4), 8.97 (dd, *J*_24_ = 1.8 Hz, *J*_23_ = 4.8 Hz, 1H, H-2). ^13^C-NMR (CDCl_3_, 150 MHz) δ 22.7 (CH_2_), 22.8 (CH_2_), 23.1 (2 × CH_2_), 24.4 (CH_2_), 24.5 (CH_2_), 31.6 (CH_2_), 31.7 (CH_2_), 34.5 (2 × CH_2_), 80.6 (2 × OCH_2_), 126.3 (C-3), 126.8 (C-4a), 133.2 (C-4), 146.0 (C-7), 146.9 (C-6), 148.3 (C-8a), 153.2 (C-2), 179.8 (C-8), 180.5 (C-5). EI MS (70 eV) *m*/*z*: 355 [M^+^] (1), 273 (12), 192 (100), 163 (34), 135 (11), 83 (18), 77 (4). IR (KBr, cm^−1^) ν_max_: 2935, 2858, 1669, 1599-1559. HR-MS (APCI) *m/z*: C_21_H_25_NO_4_ [M + H]^+^, Calcd. 356.1862; Found 356.1853.

*6,7-di(1-Methylbenzyloxy)-5,8-quinolinedione* (**18**): Yield: 57%, oil. ^1^H-NMR (CDCl_3_, 600 MHz) δ 1.69–1.71 (m, 6H, 2 × CH_3_), 1.73–1.75 (m, 6H, 2 × CH_3_), 5.93–6.10 (m, 4H, 4 × CH), 7.27–7.30 (m, 4H, Ph), 7.37–7.43 (m, 16H, Ph), 7.54–7.60 (m, 2H, 2 × H-3), 8.25–8.30 (m, 2H, 2 × H-4), 8.89–8.92 (m, 2H, 2 × H-4). ^13^C-NMR (CDCl_3_, 150 MHz) δ 22.5 (2 × CH_3_) 22.6 (2 × CH_3_), 69.0 (2 × CH), 69.3 (2 × CH), 125.3 (2 × Ph), 125.5 (4 × Ph), 126.4 (2 × Ph), 127.2 (2 × Ph), 127.3 (2 × C-3), 127.4 (4 × Ph), 127.5 (4 × Ph), 132.1 (2 × C-4a), 133.0 (2 × C-4), 144.9 (2 × Ph), 145.5 (C-7), 145.6 (C-7), 146.6 (C-6), 146.7 (C-6), 148.0 (C-8a), 148.1 (C-8a), 153.1 (2 × C-2), 179.3 (C-8), 179.4 (C-8), 180.0 (C-5), 180.1 (C-5). EI MS (70 eV) *m/z*: 399 [M^+^] (0.16), 295 (4), 279 (0.3), 191 (32), 163 (3), 105 (100), 77 (10). IR (KBr, cm^−1^) ν_max_: 3087-2899, 1684, 1680, 1669, 1663, 1601–1560. HR-MS (APCI) *m*/*z*: C_25_H_21_NO_4_ [M + H]^+^, Calcd. 400.1549; Found 400.1559.

### 3.4. Crystal Structure Determination

X-ray diffraction measurement was performed at 100 ± 1 K Before measurement, the single crystals of a good quality were preselected under a polarization microscope Oxford Cryosystem Equipment, Oxfordshire, UK). For compounds **7** and **15**, brown and green crystals with the dimensions of 0.34 × 0.29 × 0.19 mm^3^ and 0.44 × 0.08 × 0.03 mm^3^ were selected, respectively. The crystals were mounted on a glass capillary and cooled down by a cold, dry nitrogen gas stream (Oxford Cryosystem Equipment, Oxfordshire, UK). The data were collected using a SuperNova kappa diffractometer with a Sapphire3 CCD detector (Oxford Diffraction Ltd., Yarnton, UK). For the integration of the collected data, CrysAllis RED program (versions 1.171.32.29, Agilent Technologies, Santa Clara, CA, USA) was used.

#### Refinement

The solving and refining procedures were similar for both compounds. The structures were solved using direct methods with SHELXS97 software and then the solutions were refined using the SHELXL97 program [23]. The aromatic hydrogen atoms were treated as “riding” on their parent carbon atoms with d(C − H) = 0.95 Å and assigned isotropic atomic displacement parameters equal to 1.2 times the value of the equivalent atomic displacement parameters of the parent carbon atom (U_iso_(H) = 1.2U_eq_(C)) was used. The methylene H atoms were constrained to an ideal geometry with d(C − H) = 0.99 Å or d(C – H) = 0.95 Å (for terminal methylene group) and U_iso_(H) = 1.2U_eq_(C). Methyl H atoms were constrained as riding atoms, fixed to the parent atoms with distance of 0.98 Å, only torsion angles were refined and U_iso_(H) = 1.5U_eq_(C). Hydrogen atoms involved in hydrogen bonding were refined freely with U_iso_(H) = 1.2U_eq_ of their parent atom.

CCDC-1436478 and CCDC-1436479 contain the supplementary crystallographic data for this paper. These data can be obtained free of charge from the Cambridge Crystallographic Data Centre via www.ccdc.cam.ac.uk/data_request/cif.

### 3.5. Biological Study

#### 3.5.1. Cell Culture

The synthesized compounds were tested against tumor cells SNB-19 (human glioblastoma, DSMZ-German Collection of Microorganisms and Cell Cultures, Braunschweig, Germany), MDA-MB-231 (human adenocarcinoma, breast cancer; ATCC), C-32 (human melanoma; ATCC) and nontumor cells HFF-1 (normal human fibroblasts derived from foreskin, ATCC). The cultured cells were kept at 37 °C and in 5% CO_2_. The cells were seeded (103 cells/well/100 mL DMEM supplemented with FBS (Fetal Bovine Serum, Lonza) to a final concentration of 10% and streptomycin 10 mg/mL and penicillin 1000 IU/mL (Sigma), using 96-well plates (Corning).

#### 3.5.2. Analysis of the Biological Activity

The antiproliferative effect of the synthesized compounds was determined using the Cell Proliferation Reagent WST-1 assay (Roche Diagnostics, Mannheim, Germany). This colorimetric assay is based on the cleavage of the tetrazolium salt WST-1 by mitochondrial dehydrogenases in viable cells, leading to formazan formation. After an exposure to the tested compounds at concentration varying in the range 0.4–400 nM/mL, the cell viability was quantified by a cell proliferation assay. The amount of WST-1-formazan was measured at 450 nm and appropriate calculations were performed as described previously. The cytotoxic activity of the tested compound was compared to the cisplatin (positive control). The experiments were repeated in triplicate for each concentration of the tested compound. The initial concentration of the tested compounds was 1 mg/mL DMSO. Solvent control (DMSO) was included to check that the DMSO had no effect at the concentration used. Calculations of the IC_50_ values were performed using the GraphPad Prism 6 (GraphPad Software, San Diego, CA, USA).

## 4. Conclusions

The mono- and disubstituted derivatives **2**–**18** were synthesized with good yields using 6,7-dichloro-5,8-quinolinedione **1** as a starting material. The reactions of compound **1** with alcohols were performed in THF solution in the presence of potassium carbonate, leading to formation of 7-substituted derivatives. Using DMSO solvent instead of THF, we were able to obtain the 6,7-disubstituted derivatives. The molecular structures of the title compounds were confirmed by ^1^H-, ^13^C-NMR, IR and MS spectra. Furthermore, structures of 6-chloro-7-(2-cyanoethoxy)-5,8-quinolinedione **7** and 6,7-di(2,2,2-trifluoroethoxy)-5,8-quinolinedione **15** were also tested using X-ray diffraction analysis. The evaluation of the *in vitro* cytotoxic activity was carried out against three human cancer lines: C-23, SNB-19 and MDA-MB-231. Most of the synthesized compounds showed high cytotoxic activity, depending on the type of substituents and applied tumor cells.

## Supplementary Materials

Supplementary materials can be accessed at: http://www.mdpi.com/1420-3049/21/2/156/s1.

## References

[1] World Health Organization Global Health Estimates Summary. http://www.who.int.

[2] Rao K.V., Cullen W.P. (1960). Streptonigrin, an antitumor substance. I. Isolation and characterization. Antibiot. Annu..

[3] Boger D.L., Yasuda M., Mitscher L.A., Drake S.D., Kitos P.A., Thompson S.C. (1987). Streptonigrin and lavendamycin partial structures. Probes for the minimum, potent pharmacophore of streptonigrin, lavendamycin, and synthetic quinoline-5,8-diones. J. Med. Chem..

[4] Bringmann G., Reichert Y., Kane V.V. (2004). The total synthesis of streptonigrin and related antitumor antibiotic natural products. Tetrahedron.

[5] Bringmann G., Reichert M., Hemnberger J. (2008). The absolute configuration of streptonigrin. Tetrahedron.

[6] Donohoe T.J., Jones C.R., Kornahrens A.F., Barbosa L.C., Walport L.J., Tatton M.T., O’Hagan M., Rathi A.H., Baker B.D. (2013). Total synthesis of the antitumor antibiotic (±)-streptonigrin: First- and second-generation routes for *de novo* pyridine formation using ring-closing metathesis. J. Org. Chem..

[7] Fryatt T., Goroski D.T., Nilson Z.D., Moody C.J., Beall H. (1999). Novel quinolinequinone antitumor agents: Structure-metabolism studies with NAD(P)H:quinone oxidoreductase (NQO1). Bioorg. Med. Chem. Lett..

[8] Ryu C.-K., Jeong H.J., Lee S.K., You H.-J., Chio K.U., Shim J.-Y., Heo Y.H., Lee C.-O. (2001). Effects of 6-arylamine-5,8-quinolinediones and 6-chloro 7-arylo-5,8-isoquinolinediones on NAD(P)H: Quinone oxidoreductase (NQO1) activity and their cytotoxic potential. Arch. Pharm. Res..

[9] Rhee H.K., Park H.J., Lee S.K., Lee C.-O., Choo H.-Y. (2007). Synthesis, cytotoxicity, and DNA topoisomerase II inhibitory activity of benzofuroquinolinediones. Bioorg. Med. Chem..

[10] Leteurtre F., Kohlhagen G., Pommier Y. (1994). Streptonigrin induced topoisomerase II sites exhibit base preferences in the middle of the enzym stragger. Biochem. Biophys. Pres. Commun..

[11] Kim Y.-S., Park S.-Y., Lee H.-J., Suh M.-E., Schollmeyer D., Lee C.-O. (2003). Synthesis and cytotoxicity of 6,11-dihydro-pyrido- and 6,11-dihydro-benzo[2,3-*b*]phenazine-6,11-dione derivatives. Bioorg. Med. Chem..

[12] Shaikh I.A., Johnson F., Grollman A.P. (1986). Streptonigrin. 1. Structure-activity relationships among simple bicyclic analogs. Rate dependence of DNA degradation on quinone reduction potential. J. Med. Chem..

[13] Cai W., Hassani M., Karki R., Walter E.D., Koelsch K.K., Seradj H., Lineswala J.P., Mirzaei H., York Y.S., Olang F. (2010). Synthesis, metabolism and *in vitro* cytotoxicity studies on novel lavendamycin antitumor agents. Bioorg. Med. Chem..

[14] Hassani M., Cai W., Holley D.C., Lineswala J.P., Maharjan B.R., Ebrahimian G.R., Seradj H., Stocksdale M.G., Mohammadi F., Marvin C.C (2005). Novel lavendamycin analogues as antitumor agents: Aynthesis, *in vitro* cytotoxicity, structure-metabolism, and computational molecular modeling studies with NAD(P)H: Quinone oxidoreductase 1. J. Med. Chem..

[15] Lee D.M., Ko J.H., Lee K.I. (2007). Cesium carbonate-mediated reaction of dichloronaphthoquinone derivatives with *O*-nucleophiles. Monatsh. Chem..

[16] Mulchin B.J., Newton C.G., Baty J.W., Grasso C.H., Martin W.J., Walton M.C., Dangerfield E.M., Plunkett C.H., Berridge M.V., Harper J.L (2010). The anti-cancer, anti-inflammatory and tuberculostatic activities of a series of 6,7-substituted-5,8-quinolinequinones. Bioorg. Med. Chem..

[17] Jastrzebska M., Boryczka S., Kadela M., Wrzalik R., Kusz J., Nowak M. (2014). Synthesis, crystal structure and infrared spectra of new 6- and 7-propylamine-5,8-quinolinediones. J. Mol. Struct..

[18] Yoon E.Y., Choi H.Y., Shin K.J., Yoo K.H., Chi D.Y., Kim D.J. (2000). The regioselectivity in the reaction of 6,7-dihaloquinoline-5,8-diones with amine nucleophiles in various solvents. Tetrahedron Lett..

[19] Ryu C.-K., Sun Y.-J., Shim J.-Y., You H.-J., Choi K.U., Lee H. (2002). Synthesis and antyfungal activity of 6,7-bis-[*S*-(aryl)tio]-5,8-quinolinediones. Arch. Pharm. Res..

[20] Bhuiyan M.M.H., Ferdaush J., Uddin M.H. (2007). Densities and viscosities of binary mixtures of {dimethylsulfoxide + aliphatic lower alkanols (C1–C3)} at temperatures from T = 303.15 K to T = 323.15 K. J. Chem. Thermodyn..

[21] Kiefer J., Noack K., Kirchner B. (2011). Hydrogen bond in mixtures of dimethyl sulfoxide and cosolvents. Curr. Phys. Chem..

[22] Steiner T. (2002). The hydrogen bond in the solid state. Angew. Chem..

[23] Sheldrick G.M. (2008). A short history of SHELX. Acta Cryst. A.

