# Supplementary material for: Synthesis, Structure and Cytotoxic Activity of Mono- and Dialkoxy Derivatives of 5,8-Quinolinedione
Source: Molecules. 2016 Jan 27;21(2):156. doi: 10.3390/molecules21020156 (PMC6273037; doi:10.3390/molecules21020156)
Supplement: Supplementary file 1 [file molecules-21-00156-s001.docx]

**Supplementary Materials: Synthesis, Structure and Cytotoxic Activity of Mono- and Dialkoxy Derivatives of 5,8-Quinolinedione**

Monika Kadela, Maria Jastrzębska, Ewa Bębenek, Elwira Chrobak, Małgorzata Latocha,
Joachim Kusz, Maria Książek and Stanisław Boryczka

**Figure S1.** Hydrogen bonds in the crystal structure of 6-chloro-7-(2-cyanoethoxy)-5,8-quinolinedione **7**. Pink and black colors indicate molecules from neighbouring unit cells. See also Table S3.

**Figure S2.** Hydrogen bonding in crystal structure of 6,7-(2,2,2-trifloroethoxy)-5,8-quinolinedione **15**. Pink and black colors indicate molecules from neighbouring unit cells.

**Table S1.** Crystal parameters, data collection and refinement details for studied compounds.

| **Compound** | **7** | **15** |
| --- | --- | --- |
| Chemical formula | C_11_H_7_ClN_2_O_4_ | C_13_H_7_F_6_NO_4_ |
| Formula Mass | 266.64 | 355.20 |
| Crystal system | Monoclinic | Monoclinic |
| Space group | C 2/c | P 21/n |
| *a*/Å | 7.9383(2) | 15.5620(4) |
| *b*/Å | 10.3723(3) | 4.97230(10) |
| *c*/Å | 26.8258(7) | 17.1214(4) |
| *α*/° | 90.00 | 90.00 |
| *β*/° | 96.907(3) | 90.328(2) |
| *γ*/° | 90.00 | 90.00 |
| Unit cell volume/Å^3^ | 2192.76(10) | 1324.81(5) |
| No. of formula units per unit cell, *Z* | 8 | 4 |
| Temperature/K | 100(2) | 100(2) |
| Crystal size/mm^3^ | 0.34x0.29x0.19 | 0.44x0.08x0.03 |
| Radiation type | MoK\α | MoK\α |
| Absorption coefficient, *μ*/mm^-1^ | 0.357 | 0.185 |
| No. of reflections measured | 16237 | 8709 |
| No. of independent reflections | 1952 | 4328 |
| *R_int_* | 0.0352 | 0.0334 |
| Final *R_1_* values (*I* > 2*σ*(*I*)) | 0.0631 | 0.0399 |
| Final *wR*(*F*^2^) values (*I* > 2*σ*(*I*)) | 0.1551 | 0.0929 |
| Final *R_1_* values (all data) | 0.0633 | 0.0485 |
| Final *wR*(*F*^2^) values (all data) | 0.1552 | 0.0978 |
| Goodness of fit on *F*^2^ | 1.297 | 1.069 |
| CCDC number | 1436478 | 1436479 |

**Table S2.** Geometric parameters (bond length and angles) for of 6-chloro-7-(2-cyanoethoxy)-5,8-quinolinedione **7** and 6,7-(2,2,2-trifloroethoxy)-5,8-quinolinedione **15** (Å, Degree).

| **6-Chloro-7-(2-cyanoethoxy)-5,8-quinolinedione 7** | | | |
| --- | --- | --- | --- |
| Cl1-C6 | 1.72(1) | C9-C10-C11 | 111.38 |
| O1-C5 | 1.22(2) | H10A-C10-H10B | 107.99 |
| O2-C8 | 1.21(2) | H10A-C10-C11 | 109.35 |
| O3-C7 | 1.34(1) | H10B-C10-C11 | 109.35 |
| O3-C9 | 1.46(1) | N2-C11-C10 | 177.58 |
| N1-C2 | 1.33(1) | C9-O3-C7-C6 | 150.36 |
| N1-C8A | 1.34(1) | C9-O3-C7-C8 | -36.40 |
| N2-C11 | 1.14(1) | C7-O3-C9-H9A | 93.54 |
| C2-H2 | 0.95(1) | C7-O3-C9-H9B | -26.64 |
| C2-C3 | 1.39(1) | C7-O3-C9-C10 | -146.55 |
| C3-H3 | 0.95(1) | C8A-N1-C2-H2 | -179.75 |
| C3-C4 | 1.38(1) | C8A-N1-C2-C3 | 0.25 |
| C4-H4 | 0.95(1) | C2-N1-C8A-C4A | 1.74 |
| C4-C4A | 1.40(1) | C2-N1-C8A-C8 | -175.81 |

**Table S2.** *Cont.*

| **6-Chloro-7-(2-cyanoethoxy)-5,8-quinolinedione 7** | | | |
| --- | --- | --- | --- |
| C4-C5 | 1.49(1) | N1-C2-C3-H3 | 178.05 |
| C4A-C8A | 1.39(1) | N1-C2-C3-C4 | -1.95 |
| C5-C6 | 1.47(1) | H2-C2-C3-H3 | -1.95 |
| C6-C7 | 1.35(1) | H2-C2-C3-C4 | 178.05 |
| C7-C8 | 1.50(1) | C2-C3-C4-H4 | -178.31 |
| C8-C8A | 1.49(1) | C2-C3-C4-C4A | 1.69 |
| C9-H9A | 0.99(1) | H3-C3-C4-H4 | 1.69 |
| C9-H9B | 0.99(1) | H3-C3-C4-C4A | -178.31 |
| C9-C10 | 1.51(1) | C3-C4-C4A-C5 | -179.96 |
| C10-H10A | 0.99(1) | C3-C4-C4A-C8A | 0.10 |
| C10-H10B | 0.99(1) | H4-C4-C4A-C5 | 0.04 |
| C10-C11 | 1.46(1) | H4-C4-C4A-C8A | -179.89 |
| C7-O3-C9 | 119.55 | C4-C4A-C5-O1 | 0.95 |
| C2-N1-C8A | 116.48 | C4-C4A-C5-C6 | 179.52 |
| N1-C2-H2 | 118.24 | C8A-C4A-C5-O1 | -179.12 |
| N1-C2-C3 | 123.52 | C8A-C4A-C5-C6 | -0.54 |
| H2-C2-C3 | 118.24 | C4-C4A-C8A-N1 | -1.92 |
| C2-C3-H3 | 120.30 | C4-C4A-C8A-C8 | 175.54 |
| C2-C3-C4 | 119.40 | C5-C4A-C8A-N1 | 178.14 |
| H3-C3-C4 | 120.30 | C5-C4A-C8A-C8 | -4.40 |
| C3-C4-H4 | 120.67 | O1-C5-C6-CL1 | -0.71 |
| C3-C4-C4A | 118.65 | O1-C5-C6-C7 | 176.74 |
| H4-C4-C4A | 120.67 | C4A-C5-C6-CL1 | -179.26 |
| C4-C4A-C5 | 121.21 | C4A-C5-C6-C7 | -1.80 |
| C4-C4A-C8A | 117.40 | CL1-C6-C7-O3 | -0.50 |
| C5-C4A-C8A | 121.39 | CL1-C6-C7-C8 | -173.73 |
| O1-C5-C4A | 120.69 | C5-C6-C7-O3 | -177.88 |
| O1-C5-C6 | 122.41 | C5-C6-C7-C8 | 8.89 |
| C4A-C5-C6 | 116.89 | O3-C7-C8-O2 | -9.83 |
| CL1-C6-C5 | 116.78 | O3-C7-C8-C8A | 173.36 |
| CL1-C6-C7 | 119.87 | C6-C7-C8-O2 | 163.42 |
| C5-C6-C7 | 123.30 | C6-C7-C8-C8A | -13.39 |
| O3-C7-C6 | 120.00 | O2-C8-C8A-N1 | 12.02 |
| O3-C7-C8 | 119.80 | O2-C8-C8A-C4A | -165.65 |
| C6-C7-C8 | 119.85 | C7-C8-C8A-N1 | -171.23 |
| O2-C8-C7 | 120.30 | C7-C8-C8A-C4A | 11.10 |
| O2-C8-C8A | 121.85 | O3-C9-C10-H10A | 54.79 |
| C7-C8-C8A | 117.76 | O3-C9-C10-H10B | 172.84 |
| N1-C8A-C4A | 124.50 | O3-C9-C10-C11 | -66.18 |
| N1-C8A-C8 | 115.87 | H9A-C9-C10-H10A | 174.71 |
| C4A-C8A-C8 | 119.58 | H9A-C9-C10-H10B | -67.24 |
| O3-C9-H9A | 110.43 | H9A-C9-C10-C11 | 53.73 |
| O3-C9-H9B | 110.43 | H9B-C9-C10-H10A | -65.12 |
| O3-C9-C10 | 106.48 | H9B-C9-C10-H10B | 52.93 |
| H9A-C9-H9B | 108.63 | H9B-C9-C10-C11 | 173.90 |
| H9A-C9-C10 | 110.43 | C9-C10-C11-N2 | -36.43 |
| H9B-C9-C10 | 110.43 | H10A-C10-C11-N2 | -157.40 |
| C9-C10-H10A | 109.35 | H10B-C10-C11-N2 | 84.55 |
| C9-C10-H10B | 109.35 |  |  |

**Table S2.** *Cont.*

| **6,7-(2,2,2-Trifloroethoxy)-5,8-quinolinedione 15** | | | |
| --- | --- | --- | --- |
| F11-C101 | 1.33(1) | F12-C101-F13 | 107.54 |
| F12-C101 | 1.32(1) | F12-C101-C91 | 112.38 |
| F13-C101 | 1.33(1) | F13-C101-C91 | 109.78 |
| F21-C102 | 1.33(1) | F21-C102-F22 | 107.87 |
| F22-C102 | 1.33(1) | F21-C102-F23 | 106.88 |
| F23-C102 | 1.33(1) | F21-C102-C92 | 110.60 |
| O1-C5 | 1.22(2) | F22-C102-F23 | 106.57 |
| O2-C8 | 1.21(2) | F22-C102-C92 | 112.39 |
| O31-C7 | 1.34(1) | F23-C102-C92 | 112.24 |
| O31-C91 | 1.42(1) | C91-O31-C7-C6 | -168.76 |
| O32-C6 | 1.37(1) | C91-O31-C7-C8 | 12.40 |
| O32-C92 | 1.42(1) | C7-O31-C91-H91A | 63.06 |
| N1-C2 | 1.34(1) | C7-O31-C91-H91B | -58.51 |
| N1-C8A | 1.35(1) | C7-O31-C91-C101 | -177.72 |
| C2-H2 | 0.95(1) | C92-O32-C6-C5 | 117.55 |
| C2-C3 | 1.38(1) | C92-O32-C6-C7 | -67.20 |
| C3-H3 | 0.95(1) | C6-O32-C92-H92A | 134.54 |
| C3-C4 | 1.38(1) | C6-O32-C92-H92B | 15.57 |
| C4-H4 | 0.95(1) | C6-O32-C92-C102 | -104.94 |
| C4-C4A | 1.39(1) | C8A-N1-C2-H2 | -179.50 |
| C4A-C5 | 1.48(1) | C8A-N1-C2-C3 | 0.50 |
| C4A-C8A | 1.39(1) | C2-N1-C8A-C4A | -0.81 |
| C5-C6 | 1.47(1) | C2-N1-C8A-C8 | 178.36 |
| C6-C7 | 1.35(1) | N1-C2-C3-H3 | 179.98 |
| C7-C8 | 1.49(1) | N1-C2-C3-C4 | -0.02 |
| C8-C8A | 1.50(1) | H2-C2-C3-H3 | -0.02 |
| C91-H91A | 0.99(1) | H2-C2-C3-C4 | 179.98 |
| C91-H91B | 0.99(1) | C2-C3-C4-H4 | 179.81 |
| C91-C101 | 1.49(1) | C2-C3-C4-C4A | -0.19 |
| C92-H92A | 0.99(1) | H3-C3-C4-H4 | -0.19 |
| C92-H92B | 0.99(1) | H3-C3-C4-C4A | 179.81 |
| C92-C102 | 1.49(1) | C3-C4-C4A-C5 | -178.98 |
| C7-O31-C91 | 124.77 | C3-C4-C4A-C8A | -0.09 |
| C6-O32-C92 | 117.51 | H4-C4-C4A-C5 | 1.02 |
| C2-N1-C8A | 116.40 | H4-C4-C4A-C8A | 179.91 |
| N1-C2-H2 | 118.10 | C4-C4A-C5-O1 | -3.04 |
| N1-C2-C3 | 123.80 | C4-C4A-C5-C6 | 177.07 |
| H2-C2-C3 | 118.10 | C8A-C4A-C5-O1 | 178.09 |
| C2-C3-H3 | 120.40 | C8A-C4A-C5-C6 | -1.80 |
| C2-C3-C4 | 119.20 | C4-C4A-C8A-N1 | 0.62 |
| H3-C3-C4 | 120.40 | C4-C4A-C8A-C8 | -178.50 |
| C3-C4-H4 | 120.80 | C5-C4A-C8A-N1 | 179.51 |
| C3-C4-C4A | 118.39 | C5-C4A-C8A-C8 | 0.39 |
| H4-C4-C4A | 120.81 | O1-C5-C6-O32 | -3.14 |
| C4-C4A-C5 | 120.87 | O1-C5-C6-C7 | -178.32 |
| C4-C4A-C8A | 118.48 | C4A-C5-C6-O32 | 176.75 |
| C5-C4A-C8A | 120.65 | C4A-C5-C6-C7 | 1.58 |
| O1-C5-C4A | 121.78 | O32-C6-C7-O31 | 6.33 |
| O1-C5-C6 | 120.62 | O32-C6-C7-C8 | -174.81 |
| C4A-C5-C6 | 117.59 | C5-C6-C7-O31 | -178.77 |

**Table S2.** *Cont.*

| **6,7-(2,2,2-Trifloroethoxy)-5,8-quinolinedione 15** | | | |
| --- | --- | --- | --- |
| O32-C6-C5 | 115.62 | C5-C6-C7-C8 | 0.09 |
| O32-C6-C7 | 121.38 | O31-C7-C8-O2 | -2.71 |
| C5-C6-C7 | 122.82 | O31-C7-C8-C8A | 177.27 |
| O31-C7-C6 | 115.82 | C6-C7-C8-O2 | 178.52 |
| O31-C7-C8 | 123.13 | C6-C7-C8-C8A | -1.51 |
| C6-C7-C8 | 121.05 | O2-C8-C8A-N1 | 2.04 |
| O2-C8-C7 | 121.65 | O2-C8-C8A-C4A | -178.77 |
| O2-C8-C8A | 121.43 | C7-C8-C8A-N1 | -177.94 |
| C7-C8-C8A | 116.92 | C7-C8-C8A-C4A | 1.25 |
| N1-C8A-C4A | 123.73 | O31-C91-C101-F11 | -56.22 |
| N1-C8A-C8 | 115.33 | O31-C91-C101-F12 | 64.72 |
| C4A-C8A-C8 | 120.94 | O31-C91-C101-F13 | -175.65 |
| O31-C91-H91A | 111.08 | H91A-C91-C101-F11 | 63.00 |
| O31-C91-H91B | 111.08 | H91A-C91-C101-F12 | -176.06 |
| O31-C91-C101 | 103.39 | H91A-C91-C101-F13 | -56.43 |
| H91A-C91-H91B | 109.05 | H91B-C91-C101-F11 | -175.43 |
| H91A-C91-C101 | 111.08 | H91B-C91-C101-F12 | -54.49 |
| H91B-C91-C101 | 111.08 | H91B-C91-C101-F13 | 65.14 |
| O32-C92-H92A | 109.83 | O32-C92-C102-F21 | -178.09 |
| O32-C92-H92B | 109.83 | O32-C92-C102-F22 | -57.46 |
| O32-C92-C102 | 109.23 | O32-C92-C102-F23 | 62.64 |
| H92A-C92-H92B | 108.27 | H92A-C92-C102-F21 | -57.57 |
| H92A-C92-C102 | 109.83 | H92A-C92-C102-F22 | 63.05 |
| H92B-C92-C102 | 109.83 | H92A-C92-C102-F23 | -176.84 |
| F11-C101-F12 | 106.39 | H92B-C92-C102-F21 | 61.39 |
| F11-C101-F13 | 106.70 | H92B-C92-C102-F22 | -177.98 |
| F11-C101-C91 | 113.71 | H92B-C92-C102-F23 | -57.88 |

**Table S3.** Parameters (Å, Degree) of the hydrogen bonds for studied compounds **7** and **15**.

| **D–H···A** | **D–H** | **H···A** | **D···A** | **<(DHA)** |
| --- | --- | --- | --- | --- |
| 6-chloro-7-(2-cyanoethoxy)-5,8-quinolinedione **7** | | | | |
| C10-H10A···O2 | 0.99 | 2.44 | 3.41 | 167 |
| C3-H3···O1 | 0.95 | 2.48 | 3.22 | 135 |
| C4-H4···Cl1 | 0.95 | 2.86 | 3.81 | 172 |
| C9-H9B···N2 | 0.99 | 2.44 | 3.29 | 143 |
| C10-H10B···N1 | 0.99 | 2.58 | 3.53 | 160 |
| 6,7-(2,2,2-trifluoroethoxy)-5,8-quinolinedione **15** | | | | |
| C91-H91A···N1 | 0.99 | 2.64 | 3.42 | 135 |
| C91-H91A···O2 | 0.99 | 2.54 | 3.34 | 137 |
| C4-H4···O1 | 0.95 | 2.35 | 3.21 | 148 |
| C3-H3···F23 | 0.95 | 2.61 | 3.34 | 133 |
